# Packaging free software chemistry programs in Debian GNU/Linux: past, present and future

**DOI:** 10.1186/1758-2946-4-S1-F4

**Published:** 2012-05-01

**Authors:** Michael Banck

**Affiliations:** 1 Department of Chemistry, Technische Universität München, Lichtenbergstrasse 4, D-85747 Garching, Germany; 2 Debian Project

## 

Started in 1993, the Debian project is one of the oldest Free Software projects. Due to its volunteer nature, specialists from all fields contribute to the Debian GNU/Linux distribution, which includes more than 30000 packages. The Debian packaging policy, its advanced package management system and the conservative release process lead to a stable basis which is ideal for customized environments like scientific research.

The Debichem project [1] has been packaging and maintaining chemical software compliant with the Debian Free Software Guidelines (DFSG) [2] since 2006. Currently, 35 Free Software package are directly maintained by the Debichem team and 10 more are part of Debichem but maintained by others.

At the core of Debichem are the cheminformatics packages OpenBabel [3], CDK [4] and RDKit [5]. They provide file format conversion, 3D coordinate generation, molecular descriptors and fingerprints, stereochemistry prediction, conformation generation and searching, forcefields and more.

Besides those, a variety of 2D/3D visualization and molecular modelling programs, as well as ab initio, semi-empirical and molecular dynamics codes are packaged by the Debichem in Debian.

Future work will include packaging of cinfony [6], a python module which presents a common API over OpenBabel, CDK and RDKit.

## References

[B1] http://alioth.debian.org/projects/debichem

[B2] http://www.debian.org/social_contract#guidelines

[B3] O'Boyle NM Banck M James CA Morley C Vandermeersch T Hutchison GR Open Babel: An Open Chemical Toolbox J Cheminf 2011 3 33 10.1186/1758-2946-3-33 PMC3198950 21982300

[B4] Steinbeck C Han Y Kuhn S Horlacher O Luttmann E Willighagen EL The Chemistry Development Kit (CDK): An Open-Source Java Library for Chemo- and Bioinformatics J Chem Inf Comput Sci 2003 43 2 493 500 10.1021/ci025584y 12653513 PMC4901983

[B5] http://rdkit.org/

[B6] O'Boyle NM Hutchison GR Cinfony – combining Open Source cheminformatics toolkits behind a common interface Chem Cent J 2008 2 24 10.1186/1752-153X-2-24 19055766 PMC2646723

